# Diagnostic Delay in Slipped Capital Femoral Epiphysis: A Single-Centre Study in Switzerland

**DOI:** 10.3390/jcm15187294

**Published:** 2026-09-19

**Authors:** Wassim Ben Abdennebi, Andreas Tsoupras, Viola Sbampato, Romain Dayer, Giacomo De Marco, Oscar Vazquez, Christina Steiger, Anne Tabard-Fougère, Dimitri Ceroni

**Affiliations:** Paediatric Orthopaedics and Traumatology Unit, Geneva University Hospitals, University of Geneva, 1205 Geneva, Switzerland

**Keywords:** hip, slipped capital femoral epiphysis, diagnosis delay, referral, radiographic severity, surgical treatment

## Abstract

**Background/Objectives**: Slipped capital femoral epiphysis (SCFE) is frequently diagnosed after substantial delay, which may be associated with greater deformity and more complex treatment. We aim to describe diagnostic delay in a Swiss tertiary-care cohort and examine its associations with radiographic severity and surgical treatment. **Methods**: We retrospectively reviewed patients younger than 16 years treated for SCFE between 2000 and 2024. Patient-level demographic characteristics were distinguished from hip-level clinical, radiographic, and treatment variables, and hip-level regression models used patient-clustered robust standard errors. **Results**: The cohort comprised 63 patients and 71 affected hips, including 8 patients with bilateral SCFE. Diagnostic delay was 0–3 weeks for 8 hips (11%), 3–12 weeks for 34 (48%), and >12 weeks for 29 (41%). Mean Southwick angle was 35.7° (SD 19.6) and was greater in the >12-week group. In an exploratory multivariable clustered logistic model adjusted for Southwick angle, age, and surgery year, each additional week of diagnostic delay was associated with higher odds of osteotomy (OR 1.11, 95% CI 1.05–1.17; *p* < 0.001). The exploratory ROC AUC was 0.903 (patient-cluster bootstrap 95% CI 0.808–0.969). **Conclusions**: Substantial diagnostic delay was observed in this Swiss tertiary-care cohort and was associated with radiographic severity and surgical treatment. These associations remain observational and should not be interpreted causally.

## 1. Introduction

Slipped capital femoral epiphysis (SCFE) is one of the most important hip disorders of adolescence. Its prevalence has been reported to range from approximately 0.71 to 10.8 per 100,000 children [1,2], with excess body weight and obesity among the recognised epidemiological associations [2]. The typical age at presentation is approximately 12.7–13.5 years for boys and 11.2–12 years for girls, with a male predominance [2].

The most common symptoms of SCFE are limping and pain, which may be localised to the hip or groin or referred to the knee [3]. An antalgic gait may impair weight bearing, particularly in unstable SCFE [3,4]. On examination, limited internal hip rotation and obligatory external rotation during passive hip flexion are characteristic clinical findings [3].

Despite these indicators, SCFE is frequently misdiagnosed or diagnosed late by primary care professionals, including general practitioners, paediatricians, and emergency medicine clinicians [5,6,7,8]. Diagnostic delay is concerning because greater slip deformity is associated with poorer long-term clinical and radiological outcomes and may contribute to early degenerative hip disease [4,5].

Several studies have evaluated delayed SCFE diagnosis in primary care, quantified the duration of diagnostic delay, and examined contributing factors [5,6,7,8,9,10]. Reported delays commonly range from approximately 8 to 17 weeks [6,8]. Socioeconomic disadvantage, insurance status, and other barriers to healthcare access have been associated with diagnostic delay in some settings [9,10]. Switzerland has mandatory basic health insurance and broad availability of medical care. These system-level characteristics provide the healthcare context for the present cohort but do not establish that individual patients were free from socioeconomic, organisational, geographic, or other barriers to care.

The primary objective was to quantify diagnostic delay in this single-centre Swiss tertiary-care cohort and to describe the clinical service associated with referral and final diagnosis. Secondary objectives were to examine the associations between diagnostic delay, radiographic slip severity, and surgical treatment. Switzerland’s mandatory basic health insurance and broad availability of medical care provide the healthcare context in which the cohort was studied; however, healthcare coverage was not considered as an explanatory variable because no comparison group or patient-level measures of healthcare access were available.

## 2. Materials and Methods

### 2.1. Participants

After ethics approval from the local Ethics Committee (CCER 2024-02105), we conducted a retrospective review of the medical records of patients younger than 16 years admitted to our institution for SCFE between January 2000 and December 2024. The study was reported in accordance with the STROBE checklist. Because this was a single-centre retrospective study without a comparison group or patient-level measurements of socioeconomic and access barriers, the study was designed to describe diagnostic delay rather than to estimate the causal effect of healthcare access.

### 2.2. Clinical Assessment and Diagnosis

Standard demographic and morphological parameters (sex, age, ethnicity, height, weight, and BMI) were collected. To limit bias due to age and sex, BMI was interpreted using age- and sex-specific z-scores [11], and body-weight status was classified using World Health Organization cut-offs [12].

Clinical variables included the interval from reported symptom onset to final diagnosis, the service associated with referral/final diagnosis, affected side, and clinical presentation. Diagnostic delay and clinical presentation were treated as distinct variables. Clinical presentation was classified by the treating surgeon according to symptom duration and clinical evolution. Acute presentation was defined as a symptom duration of ≤3 weeks, whereas chronic presentation was defined as symptoms lasting >3 weeks without an acute exacerbation. Acute-on-chronic presentation was defined as symptoms lasting >3 weeks with subsequent progressive acute worsening, potentially resulting in inability to bear weight. Among patients with bilateral SCFE, simultaneous and sequential presentations were distinguished. For simultaneous bilateral presentations, both hips shared the same presentation-level variables, including age, symptom onset, diagnostic delay, referral/final-diagnosis service, and surgery year, while hip-specific radiographic and treatment variables were recorded separately. For sequential bilateral presentations, age, symptom onset, diagnostic delay, referral/final-diagnosis service, surgery year, radiographic measurements, and treatment were assigned separately to each affected hip according to the corresponding episode. The available data do not provide a complete structured chronology of first-contact physician, number/dates of previous consultations, initial diagnosis, imaging and referral pathway for every patient; therefore, patient-related and provider-related components of delay could not be separated. The service associated with referral/final diagnosis was consequently analysed as a descriptive variable only and was not considered an indicator of the source of diagnostic delay.

### 2.3. Evaluation of the Treatment Pathway

In Switzerland, families generally consult either their family physician or paediatrician for health problems that do not require emergency care. For serious or urgent conditions, they typically seek care at a nearby facility providing emergency services, such as a tertiary hospital or a clinic with an emergency department. Emergency services may also be used for persistent conditions when families believe that additional specialist expertise is required. Families rarely consult a paediatric orthopaedic surgeon directly, as referrals are typically made by a paediatrician or an emergency physician.

### 2.4. Radiographic Assessment and Outcomes

#### 2.4.1. Primary Outcomes

The primary outcome was diagnostic delay, defined as the interval between reported symptom onset and the date on which SCFE was established. The delay was analysed both continuously and in the prespecified categories: 0–3 weeks, 3–12 weeks, and >12 weeks.

#### 2.4.2. Secondary Outcomes

Secondary outcomes included radiographic slip severity and surgical management. The Southwick slip angle was measured on Lauenstein (frog-leg lateral) radiographs by a single senior paediatric orthopaedic surgeon involved in the surgical management. The angle was defined between a line perpendicular to the epiphyseal base and the longitudinal axis of the femur. To quantify the slip in unilateral SCFE, the corresponding angle measured on the unaffected contralateral side was subtracted from the angle of the affected side. Severity was categorised as mild (<30°), moderate (30–60°), or severe (>60°). In bilateral cases, each affected hip was analysed separately using a theoretical reference value of 12° for the contralateral Southwick angle, as no unaffected contralateral hip was available for comparison. No repeated measurements or formal inter- or intra-observer reliability analysis were performed.

### 2.5. Statistical Analysis

Analyses were performed using R software (version 4.4.1; R Foundation for Statistical Computing, Vienna, Austria), RStudio (version 2024.09.0; Posit Software, PBC, Boston, MA, USA), and Python (version 3.13.5; Python Software Foundation, Beaverton, OR, USA), with statsmodels (version 0.14.6), scikit-learn (version 1.8.0), and SciPy (version 1.17.0). Continuous outcomes are reported as mean (SD) [range] and categorical outcomes as *n* (%). Because some patients contributed with bilateral injury, hip-level logistic regression used sandwich/robust standard errors clustered by patient. Osteotomy was coded as 1 and screw-only fixation as 0. Given the limited number of osteotomy events, treatment models were considered exploratory. Diagnostic delay was modelled continuously per additional week to avoid complete separation caused by the 0–3-week category. Southwick angle was scaled per 10° increase. Univariable models examined age, diagnostic delay, Southwick angle, and surgery year, and an exploratory multivariable model included all four variables. Linearity in the logit for continuous diagnostic delay and surgery year was assessed by adding quadratic terms to the multivariable model. To compare Southwick angle across the three diagnostic delay categories while accounting for bilateral hips, a Gaussian generalised estimating equation (GEE) with exchangeable working correlation was used, followed by Holm-adjusted pairwise contrasts. Exploratory ROC analysis assessed within-sample discrimination of continuous diagnostic delay for osteotomy. To account for bilateral clustering and internal optimism, 95% CIs for the AUC and the distribution of the Youden cut-point were estimated from 2000 patient-level cluster-bootstrap samples. The optimal cut-point was defined as the value maximising Youden’s J statistic [13] (sensitivity + specificity − 1). All tests were two-sided, and *p* < 0.05 was considered statistically significant.

## 3. Results

### 3.1. Demographic Characteristics

**Figure 1:**
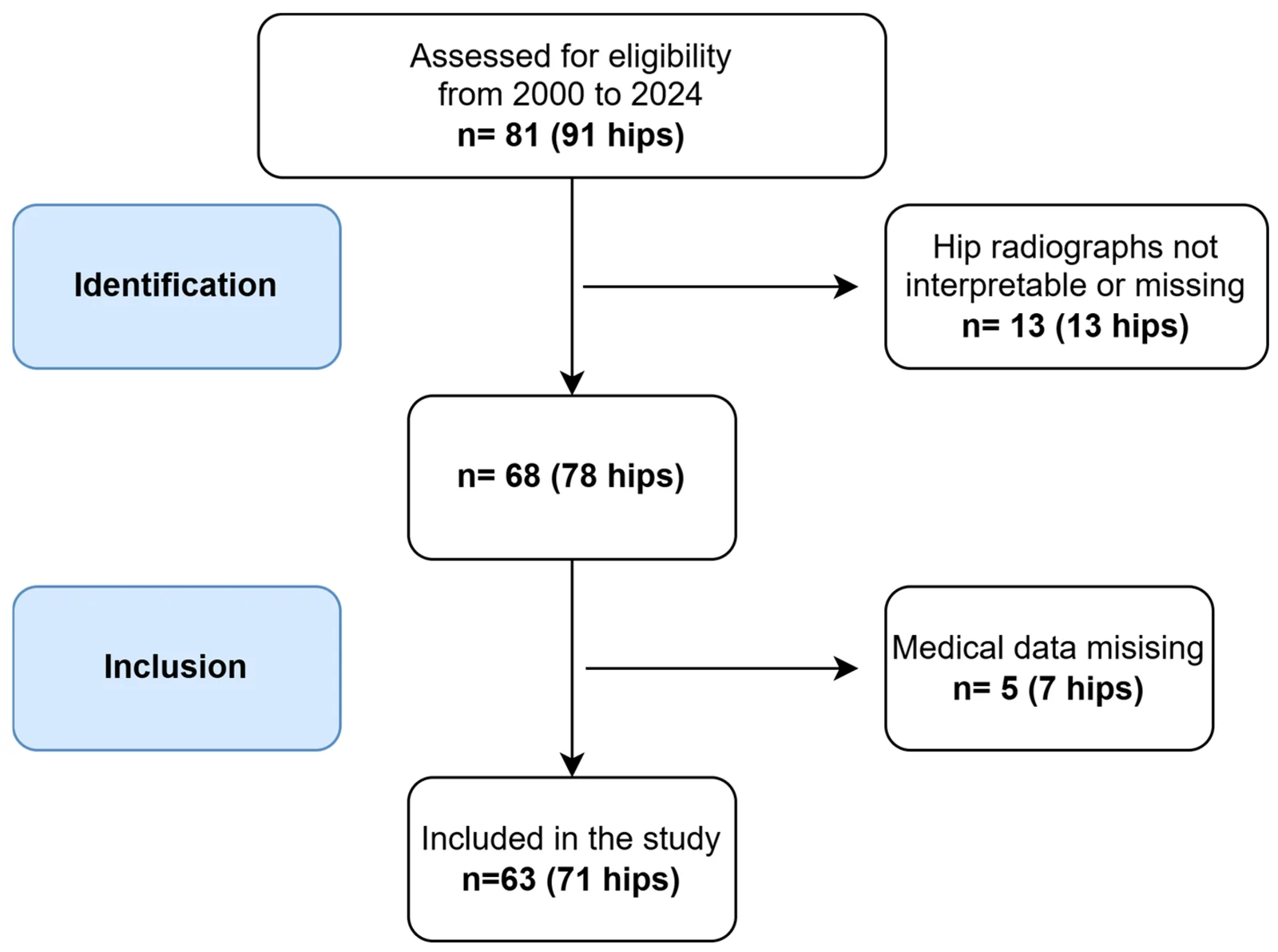
Study flow chart. Eighty-one patients treated for SCFE were screened. Eighteen patients were excluded because the imaging assessment and/or required clinical information was incomplete. The definitive study cohort therefore comprised 63 patients and 71 affected hips.

The cohort comprised 63 patients and 71 affected hips. A total of 55 patients presented with unilateral SCFE and 8 with bilateral SCFE (55 + 8 = 63 patients; 55 + 16 = 71 affected hips). Subjects with non-interpretable or missing X-rays and missing medical data were excluded from the cohort (Figure 1).

The cohort comprised 29 females (46%) and 34 males (54%), with an overall mean age of 12.2 ± 1.5 years. Regarding ethnicity, 43 patients (68%) were European (29 Swiss and 14 other European), 10 (16%) African, 7 (11%) South American, and 3 (5%) Asian. Body-weight status was available for 60 patients: 30 (50%) were obese, 17 (28%) overweight, 12 (20%) normal weight, and 1 (2%) underweight. Patient characteristics are summarised in Table 1.

### 3.2. Speciality of the Clinician Establishing the Diagnosis and Diagnostic Delay

At hip-record level, the service associated with referral/final diagnosis was paediatrics in 23/71 cases (32%), emergency medicine in 39/71 (55%) and orthopaedics in 9/71 (13%). All 23 paediatric-associated hip records had a diagnostic delay > 3 weeks; 13/23 (57%) had a delay > 12 weeks. These data are descriptive and do not identify the first-contact clinician or the stage of the care pathway at which delay occurred. Because the chronology and content of previous consultations were not systematically available, these findings cannot identify which stage of the care pathway contributed to diagnostic delay or attribute delay to a particular medical speciality.

Diagnosis occurred within 0–3 weeks in 8 hip-level cases (11%), within 3–12 weeks in 34 (48%), and after >12 weeks in 29 (41%). Mean diagnostic delay was 114.9 days (SD 124.3; range 7–365).

### 3.3. Characteristics of SCFE

**Figure 2:**
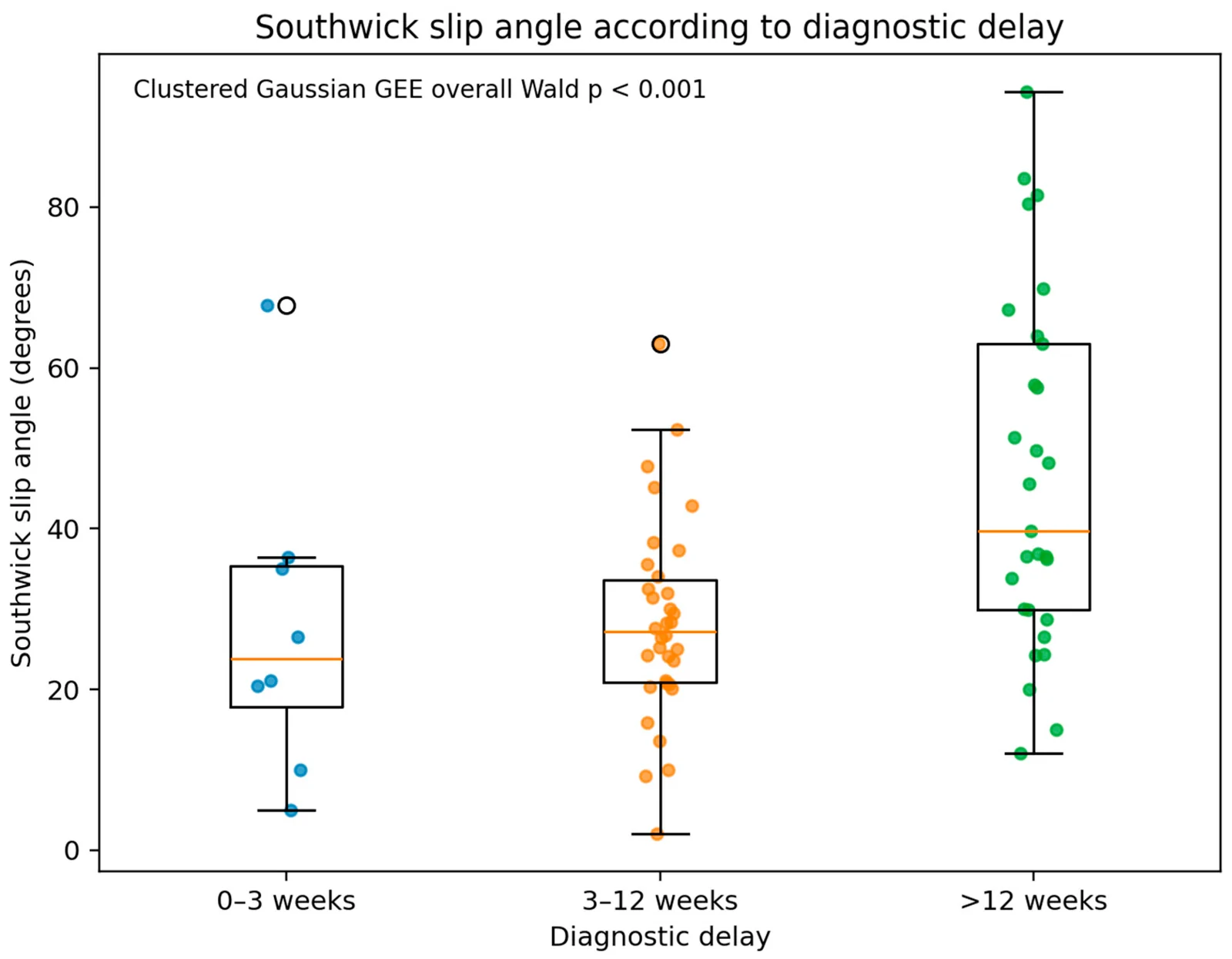
Southwick slip angle according to diagnostic delay (*n* = 71 affected hips). Boxes show the distribution within the 0–3-week, 3–12-week, and >12-week groups; individual hip-level observations are displayed. A Gaussian GEE clustered by patient showed an overall association between delay category and Southwick angle (Wald *p* < 0.001). Holm-adjusted pairwise comparisons were significant for >12 weeks versus 0–3 weeks (*p* = 0.022) and >12 weeks versus 3–12 weeks (*p* < 0.001), but not for 0–3 weeks versus 3–12 weeks (*p* = 0.884).

In the cohort, 47/71 affected hips were left-sided (66%), and 24/71 were right-sided (34%). Eight patients had bilateral SCFE, accounting for 16 hip records; five presented with simultaneous bilateral involvement and three developed sequential contralateral SCFE. The remaining 55 patients had unilateral disease. Clinical presentation was classified as chronic in 55/71 hips (77.5%), acute in 14/71 (19.7%), and acute-on-chronic in 2/71 (2.8%). Mean Southwick slip angle was 35.7° (SD 19.6; range 2.0–94.4). Slip severity comprised 34 mild hips (<30°; 47.9%), 27 moderate hips (30–60°; 38.0%), and 10 severe hips (>60°; 14.1%). Mean Southwick angle was 27.8° in the 0–3-week group, 28.4° in the 3–12-week group, and 46.4° in the >12-week group. A patient-clustered Gaussian GEE showed an overall association between diagnostic delay category and Southwick angle (Wald *p* < 0.001). After Holm correction, the >12-week group had higher Southwick angles than both the 0–3-week group (adjusted *p* = 0.022) and the 3–12-week group (adjusted *p* < 0.001), whereas the two shorter-delay groups did not differ (adjusted *p* = 0.884). Figure 2 summarises the distribution of Southwick slip angle across diagnostic delay categories of the cohort. The marked increase in radiographic severity was concentrated in hips diagnosed after >12 weeks, whereas the 0–3-week and 3–12-week groups had similar mean Southwick angles.

### 3.4. Surgical Treatment and Diagnostic Delay

In our cohort, treatment comprised screw-only fixation in 49/71 hips (69%) and osteotomy in 22/71 (31%). Osteotomy occurred in 0/8 hips diagnosed within 0–3 weeks, 3/34 diagnosed within 3–12 weeks, and 19/29 diagnosed after >12 weeks. Because the categorical delay variable showed complete separation, regression used continuous delay (per additional week). In univariable patient-clustered logistic regression, longer delay was associated with osteotomy (OR 1.12 per week, 95% CI 1.05–1.19; *p* < 0.001), as was a larger Southwick angle (OR 2.03 per 10° increase, 95% CI 1.25–3.29; *p* = 0.004). Age (OR 1.34 per year, 95% CI 0.93–1.95; *p* = 0.117) and surgery year (OR 1.04 per year, 95% CI 0.93–1.15; *p* = 0.506) were not significant. In the exploratory multivariable model including diagnostic delay, Southwick angle, age and surgery year, diagnostic delay remained associated with osteotomy (adjusted OR 1.11 per week, 95% CI 1.05–1.17; *p* < 0.001), as did Southwick angle (adjusted OR 2.00 per 10°, 95% CI 1.24–3.20; *p* = 0.004), whereas age and surgery year were not significant (Table 2). Exploratory assessment of functional form showed no evidence of departure from linearity in the logit for diagnostic delay (quadratic term, *p* = 0.783) or surgery year (quadratic term, *p* = 0.961). Clinical presentation was not added to the multivariable model because only two hips were classified as acute-on-chronic and only 22 osteotomies occurred, making further model expansion inappropriate in this event-limited exploratory analysis.

**Figure 3:**
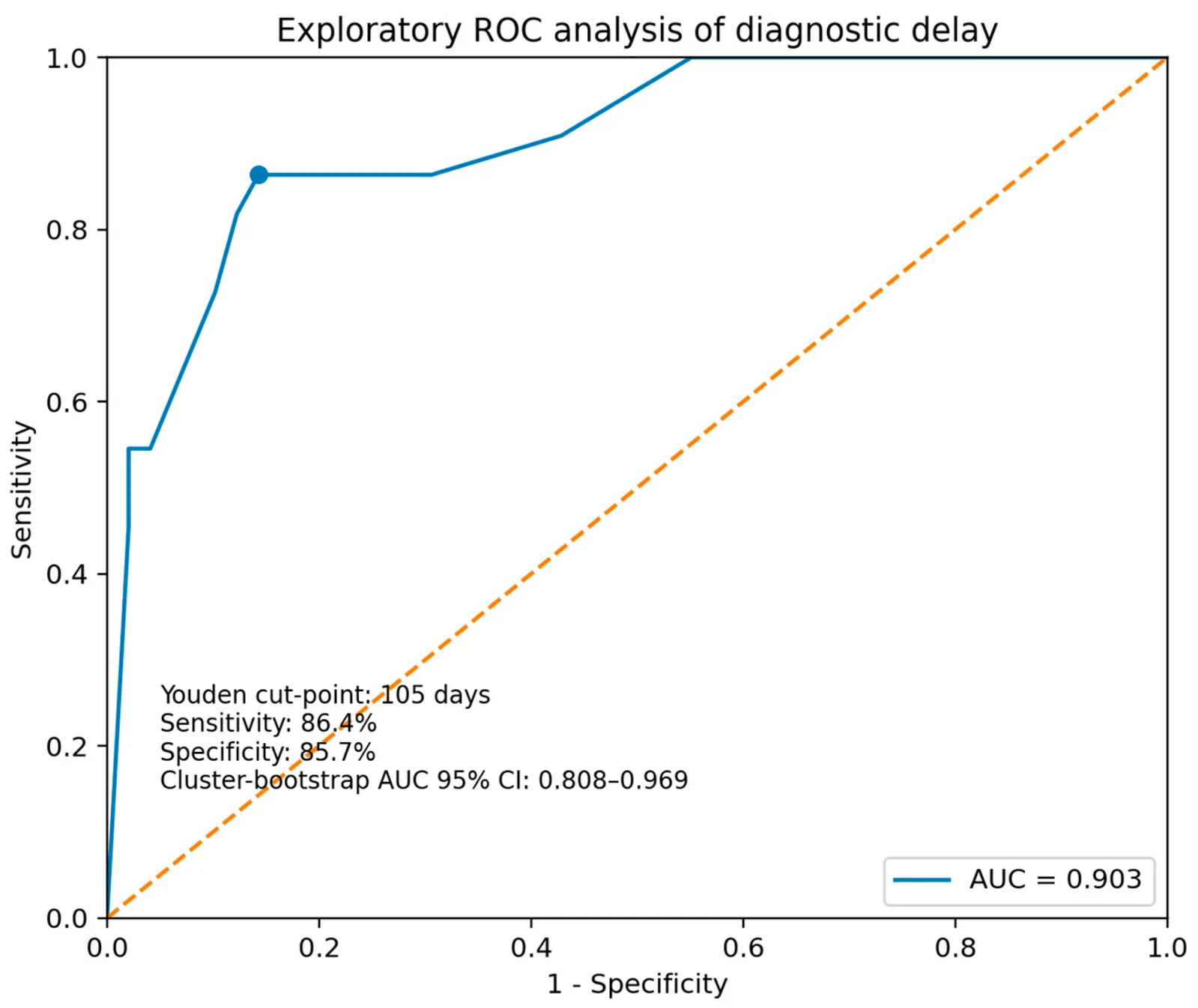
Exploratory ROC analysis of continuous diagnostic delay for treatment type (osteotomy versus screw-only fixation) (*n* = 71 hips). AUC = 0.903; patient-cluster bootstrap 95% CI 0.808–0.969. The Youden cut-point was 105 days (sensitivity 86.4%, specificity 85.7%); the bootstrap 95% percentile range for the optimal cut-point was 90–165 days. The cut-point was derived and evaluated in the same cohort and is not a validated treatment threshold.

Continuous diagnostic delay showed high within-sample discrimination for treatment type (AUC = 0.903; Figure 3). The Youden cut point was 105 days, corresponding to a sensitivity of 86.4% and specificity of 85.7%. In 2000 patient-level cluster-bootstrap samples, the AUC 95% interval was 0.808–0.969, and the bootstrap distribution of the optimal cut-point had a 2.5th–97.5th percentile range of 90–165 days. These estimates indicate substantial uncertainty around the cut-point; therefore, 105 days is presented only as an exploratory within-sample marker and not as a validated clinical threshold or an individual probability of osteotomy.

## 4. Discussion

Switzerland has mandatory basic health insurance and broad availability of medical care. These characteristics provide the healthcare context in which this cohort was studied, but healthcare coverage was not measured as an exposure. Because the study had no healthcare system comparison group and did not collect patient-level measures of socioeconomic status, travel distance, waiting times, insurance-related barriers, or other direct measures of healthcare access, the findings cannot determine whether the Swiss healthcare context shortened, prolonged, or otherwise influenced diagnostic delay.

The mean patient age was 12.2 years, consistent with the expected age range for SCFE [2,5]. Body-weight status was available for 60 of 63 patients: 50% were obese, 28% overweight, 20% normal weight, and 2% underweight. These findings reinforce that typical age and body morphology can support clinical suspicion but should not be used in isolation to exclude SCFE, particularly when compatible hip, groin, thigh, or knee symptoms are present [2,3].

At the hip-record level, 89% of cases were diagnosed more than 3 weeks after symptom onset: 48% at 3–12 weeks and 41% after >12 weeks. Mean diagnostic delay was 114.9 days (approximately 16.4 weeks), within the broad range reported in previous studies [5,6,8]. Substantial diagnostic delay was therefore observed in this Swiss cohort, but cross-study comparisons cannot establish an effect of healthcare coverage on diagnostic timeliness.

The service associated with referral or final diagnosis was recorded descriptively. Emergency medicine was the most frequently recorded service, followed by paediatrics and orthopaedics. However, these data represent the service documented at referral or final diagnosis and do not reconstruct the preceding healthcare pathway. Because the chronology and content of previous consultations were not systematically available, these findings cannot identify which stage of the care pathway contributed to diagnostic delay or attribute delay to a particular medical speciality.

Longer diagnostic delay was associated with both greater radiographic severity and a higher frequency of osteotomy. In the exploratory multivariable analysis, diagnostic delay remained associated with osteotomy after adjustment for Southwick angle, age, and surgery year (adjusted OR 1.11 per additional week, 95% CI 1.05–1.17), while Southwick angle was also associated with treatment (adjusted OR 2.00 per 10° increase, 95% CI 1.24–3.20). This association should not be interpreted as evidence that diagnostic delay independently determines the choice of surgical treatment. Treatment selection was not protocolised solely according to these variables and may also have been influenced by clinical stability, acute-on-chronic presentation, surgeon judgement, anatomical characteristics, and other factors not captured in the model. Residual confounding and treatment selection bias therefore remain possible. Surgery year was not associated with osteotomy, although this does not exclude more complex temporal effects. The ROC analysis showed high within-sample discrimination (AUC 0.903), but bootstrap uncertainty around the optimal cut-point was substantial; consequently, the 105-day value should not be interpreted as a validated clinical threshold or as evidence that earlier diagnosis would necessarily have prevented osteotomy.

These findings support the general need to maintain awareness of SCFE among clinicians who assess children and adolescents with hip, groin, thigh, or knee symptoms and to obtain appropriate radiographic assessment, including lateral views when clinically suitable. Prospective studies incorporating detailed referral pathways, patient-level access variables, clinical stability, and treatment selection factors are needed to determine which factors contribute to diagnostic delay and surgical management.

This study has several limitations. Its retrospective, single-centre design is susceptible to incomplete documentation, recall error, missing data, and selection bias. As a tertiary paediatric orthopaedic centre, the institution may receive disproportionately severe or complex cases, potentially increasing observed slip severity and osteotomy frequency. A total of 18 of the 81 screened patients were excluded because imaging assessment and/or required clinical information was incomplete, which may have introduced selection bias. Hip-level analyses account for within-patient clustering, but only 22 osteotomies occurred, limiting model complexity, stability, and precision. The multivariable treatment analysis should therefore be regarded as exploratory rather than confirmatory. Although exploratory quadratic-term analyses showed no evidence of departure from linearity in the logit for diagnostic delay or surgery year, the limited number of outcome events reduces the power to detect nonlinear relationships. Although Southwick angle, age, and surgery year were included, clinical stability according to the Loder classification was not systematically recorded. Residual confounding by factors influencing treatment selection, such as slip stability, acute-on-chronic presentation, surgeon judgement, anatomical characteristics, and surgeon preference, therefore remains possible. The 2000–2024 inclusion period may also encompass changes in imaging, referral, and operative practice. Surgery year was included as a continuous covariate and was not significantly associated with treatment, but this does not exclude more complex temporal effects. The ROC cut-point was derived and evaluated in the same cohort and therefore remains vulnerable to optimism despite patient-level cluster bootstrap. Finally, Southwick measurements were performed once by a single senior paediatric orthopaedic surgeon involved in surgical management; no repeated measurements or formal inter- or intra-observer reliability analysis was performed, and measurement reproducibility could therefore not be quantified.

## 5. Conclusions

Substantial diagnostic delays of SCFE were observed in this single-centre Swiss cohort. Longer delay was associated with greater radiographic severity and remained associated with osteotomy in an exploratory patient-clustered analysis adjusted for Southwick angle, age, and surgery year. These findings do not establish causation, identify a specific physician speciality as responsible for delay, or prove that earlier diagnosis would have prevented osteotomy. The results support continued awareness of SCFE and timely appropriate imaging, while larger prospective studies with detailed care pathways, patient-level access measures, clinical-stability data, and treatment selection factors are needed to confirm these associations.

## Figures and Tables

**Table 1:** Population characteristics.

	N = 63 Patients (71 Hips)
Female, *n* (%)	29 (46%)
Male, *n* (%)	34 (54%)
Age at diagnosis, year	12.2 (1.5) [8.8–15.8]
Prepubertal, *n* (%)	30 (48%)
Pubertal, *n* (%)	28 (44%)
Post pubertal, *n* (%)	5 (8%)
Height, cm	156.3 (14.3) [97.0–181.0]
Weight, kg	60.9 (15.3) [28.0–93.0]
Body weight status	
Obese, *n* (%)	30/60 (50%)
Overweight, *n* (%)	17/60 (28%)
Normal weight, *n* (%)	12/60 (20%)
Underweight, *n* (%)	1/60 (2%)
Ethnicity	
European, *n* (%)	43 (68%)
African, *n* (%)	10 (16%)
South American, *n* (%)	7 (11%)
Asian, *n* (%)	3 (5%)
71 Hips	
Left hip only, *n* hips (%)	47 (66%)
Right hip only, *n* hips (%)	24 (34%)
Bilateral-case accounting	8 bilateral patients (16 hips): 5 simultaneous, 3 sequential
Clinical presentation (*)	
Chronic, *n* (%)	55/71 (77.5%)
Acute, *n* (%)	14/71 (19.7%)
Acute-on-chronic, *n* (%)	2/71 (2.8%)
Delay between symptoms and diagnosis (*)	
Mean (SD) [range], days	114.9 (124.3) [7.0–365.0]
0–3 weeks, *n* (%)	8 (11%)
3–12 weeks, *n* (%)	34 (48%)
>12 weeks, *n* (%)	29 (41%)
Delay between diagnosis and surgery (*)	
Mean (SD) [range], days	10.7 (31.4) [0.0–191.0]
0–3 weeks, *n* (%)	68 (96%)
3–12 weeks, *n* (%)	1 (1%)
>12 weeks, *n* (%)	2 (3%)
Service associated with referral/final diagnosis by hip (*)	
Emergency medicine, *n* (%)	39 (55%)
Paediatrics, *n* (%)	23 (32%)
Orthopaedics, *n* (%)	9 (13%)
Type of surgery (*)	
Screws only, *n* (%)	49 (69%)
Osteotomy, *n* (%)	22 (31%)
Southwick slip angle, degrees	35.7 (19.6) [2.0–94.4]
Slip Severity	
Mild, *n* (%)	34 (47.9%)
Moderate, *n* (%)	27 (38.0%)
Severe, *n* (%)	10 (14.1%)

(*) Hip-level variables are reported for a total of 71 affected hips. Patient-level demographic variables are reported for 63 unique patients. Body-weight status was available for 60/63 patients; percentages for weight categories are therefore calculated using the available cases. Age-based pubertal categories were derived from sex-specific age thresholds rather than Tanner staging and may therefore misclassify biological pubertal status.

**Table 2:** Univariable and multivariable patient-clustered logistic regression for osteotomy versus screw-only fixation (*n* = 71 affected hips; 22 osteotomies).

Predictor	Unit	Univariable OR (95% CI)	*p* Value	Adjusted OR (95% CI)	*p* Value
Age	per 1 year	1.34 (0.93–1.95)	0.117	1.09 (0.76–1.57)	0.629
Diagnostic delay	per 1 week	1.12 (1.05–1.19)	<0.001	1.11 (1.05–1.17)	<0.001
Southwick slip angle	per 10°	2.03 (1.25–3.29)	0.004	2.00 (1.24–3.20)	0.004
Surgery year	per 1 year	1.04 (0.93–1.15)	0.506	0.99 (0.83–1.18)	0.908

OR = odds ratio; CI = confidence interval. Osteotomy was coded as 1 and screw-only fixation as 0. Robust sandwich standard errors were clustered by patients. The multivariable model included all four listed predictors. Diagnostic delay was modelled continuously because the categorical variable showed complete separation in the 0–3-week group.

## Data Availability

The data supporting the findings of this study are not openly available due to sensitivity concerns. They are accessible from the corresponding author upon reasonable request. The data are stored in a controlled-access repository at Geneva University Hospitals, Switzerland.

## Abbreviations

The following abbreviations are used in this manuscript:

SCFE	Slipped Capital Femoral Epiphysis
MRI	Magnetic Resonance Imaging
BMI	Body Mass Index
